# Our Ways, Your Ways, Both Ways – a multi-disciplinary collaboration to develop, embed and evaluate a model of social and emotional wellbeing care for Aboriginal and Torres Strait Islander young people who experience detention – Phase 1

**DOI:** 10.3389/fpsyt.2023.1207103

**Published:** 2023-10-20

**Authors:** Penny R. Dale, Carla Meurk, Megan Williams, Marshall Watson, Megan L. Steele, Lisa Wittenhagen, Scott Harden, Stephen Stathis, James G. Scott, Stuart Kinner, Ed Heffernan

**Affiliations:** ^1^Queensland Forensic Mental Health Service, Queensland Health, Brisbane, QLD, Australia; ^2^School of Public Health, The University of Queensland, Herston, QLD, Australia; ^3^Forensic Mental Health Group, Queensland Centre for Mental Health Research, Wacol, QLD, Australia; ^4^Girra Maa Indigenous Health Discipline, Graduate School of Health, University of Technology Sydney, Sydney, NSW, Australia; ^5^Indigepsych, Adelaide, SA, Australia; ^6^Child and Youth Mental Health Service, Queensland Children’s Hospital, South Brisbane, QLD, Australia; ^7^Child Health Research Centre, The University of Queensland, South Brisbane, QLD, Australia; ^8^Curtin School of Population Health, Curtin University, Perth, WA, Australia; ^9^Justice Health Group, Centre for Adolescent Health, Murdoch Children’s Research Institute, Melbourne, VIC, Australia; ^10^Melbourne School of Population and Global Health, University of Melbourne, Carlton, VIC, Australia; ^11^Griffith Criminology Institute, Griffith University, Brisbane, QLD, Australia

**Keywords:** youth, mental health, social and emotional wellbeing, justice, Aboriginal, Torres Strait Islander, Indigenous, first nations

## Abstract

The National Strategic Framework for Aboriginal and Torres Strait Islander Peoples’ Mental Health and Social and Emotional Wellbeing identifies building a strong Aboriginal and Torres Strait Islander led evidence-base to inform care as a key priority. Aboriginal and/or Torres Strait Islander adolescents in contact with the criminal justice system are a highly vulnerable group of Australians, with substantial unmet needs. There is limited evidence to inform culturally appropriate models of care that meet the social and emotional wellbeing needs of justice-involved Aboriginal and/or Torres Strait Islander adolescents. This project aims to develop, implement and evaluate an in-reach and community transitional model of social and emotional wellbeing care for Aboriginal and/or Torres Strait Islander adolescents (10–17 years old) who experience detention through close engagement with Aboriginal and/or Torres Strait Islander youth, Elders, researchers, practitioners and community members, and by drawing on culturally informed practice and knowledge systems. The project is based on a multi-level mixed methods design, with a strong focus on ongoing project evaluation (based on the Ngaa-bi-nya framework) and co-design. Co-design is facilitated through culturally safe and trauma informed participatory processes based on development of strong partnerships from project initiative, design, implementation and evaluation. Application of the landscape domain of the Ngaa-bi-nya framework for Aboriginal and Torres Strait Islander program evaluation will be explored in Phase one. Aboriginal and Torres Strait Islander adolescents with experience in detention will be engaged through one-on-one interviews with data collection through the Growth and Empowerment Measure (GEM) Youth (which will be adapted from the adult version and validated as part of this study), the Kessler Psychological Distress Scale (K-10), questions around alcohol and drug use, and narrative interviews exploring experience. Qualitative data will be analyzed using an inductive thematic approach, structured within the framework of the Ngaa-bi-nya landscape prompts. Quantitative data will be analyzed using descriptive statistics to provide a profile of the cohort. Findings from Phase one will be used to inform the development of a model of social and emotional wellbeing care that will be implemented and evaluated in Phase two.

## 1. Introduction

Aboriginal and Torres Strait Islander young people are disproportionately represented in the criminal justice system, they tend to have contact with the youth justice system at a younger age compared to non-Indigenous young people and they are more likely to return to youth detention before the age of 18 (1). They experience social disadvantage related to poor education, health problems, housing problems and social exclusion, with substantial unmet social, emotional and mental health and wellbeing needs. Failure to address these needs was highlighted in the 1989 Royal Commission into Aboriginal Deaths in Custody (2), the Bringing Them Home report (3) and Royal Commission into the Detention and Protection of Children in the Northern Territory (4) and remains a major challenge for the Australian Government commitment to ‘Closing the Gap’ and achieving health equity between Aboriginal and Torres Strait Islander peoples and others in Australia (5). Despite justice targets in Closing the Gap, there is limited evidence to inform culturally safe models of care that support young Aboriginal and Torres Strait Islander peoples transitioning from the youth justice system to the community, including models that meet their social and emotional wellbeing needs. There is guidance from the National Strategic Framework for Aboriginal and Torres Strait Islander Peoples’ Mental Health and Social and Emotional Wellbeing (6). This document identifies building a strong Aboriginal and Torres Strait Islander led evidence-base to inform social and emotional wellbeing and mental health care as a key priority.

For young people who experience detention, the period immediately following release from detention is a time of high vulnerability (7). Young people released from detention commonly face difficulties continuing education or training, accessing mainstream services and gaining employment, and they are at high risk of engaging in harmful substance use, and suicide and self-harming behaviors (8). Culturally safe services, based on an Aboriginal and Torres Strait Islander models of social and emotional wellbeing that strengthens empowerment and self-determination, are needed to address the unmet needs of these young people to achieve positive outcomes.

The IMHIP-Youth project aims to fill gaps in research and services to support the mental health, and social and emotional wellbeing needs, of Aboriginal and Torres Strait Islander young people (10–17 years old) who experience detention, by:

1. co-designing, implementing and evaluating an Aboriginal and Torres Strait Islander-led in-reach and community transitional model of social and emotional wellbeing care (Indigenous Mental Health Intervention Program – Youth model; ‘IMHIP-Youth’) that improves outcomes for Aboriginal and Torres Strait Islander young people who experience detention;
2. contributing to an evidence-base for social and emotional wellbeing models of care; and
3. promoting Aboriginal and Torres Strait Islander leadership and excellence in research, service delivery and clinical practice.

The research to support the development, implementation, and evaluation of the IMHIP-Youth service is structured into two phases: (Phase 1) a co-design and consultation phase; and (Phase 2) an evaluation phase. This document outlines the protocol for phase one of this research, which includes consenting processes and collection of baseline measures relevant to the collection of comparator data that will support the Phase two evaluation.

## 2. Background

Aboriginal and Torres Strait Islander peoples are the world’s oldest continuing cultures, with connections for over 60,000 years in Australia, colonized a little over 200 years ago. Prior to colonization, the 500+ nations worked interdependently with strict governance systems to maintain healthy lands, waterways and air. People’s social and emotional wellbeing and identity was inextricably linked to environmental wellbeing (9, 10), intergenerational connections and clear social roles supported by the community (11, 12). As a function of colonization, massacres, poisonings and mass incarceration removed Aboriginal and Torres Strait Islander peoples from homelands, bringing about rapid depopulation and destruction of social and health systems, with impacts continuing today (13–15). Aboriginal and Torres Strait Islander people were and often still are excluded from government decision making and program delivery (16), consistently have poorer access to mainstream services than other Australians (17) and frequently experience racism in service delivery (18) and at work (19). Health and criminal justice systems have not been able to meet Aboriginal and Torres Strait Islander staff targets of 3% (20) and access of the 97% of mainstream staff to cultural safety training required to bring about culturally responsive systems is minimal (21, 22).

Aboriginal and Torres Strait Islander people’s concept of social and emotional wellbeing is a broader concept than the mainstream clinical concept of mental illness, recognizing “the importance of connection to land, culture, spirituality, ancestry, family and community, and how these affect the individual” (14). Poor social and emotional wellbeing encompasses a range of problems that can result from unresolved grief and loss, trauma and abuse, violence, removal from family, substance misuse, family breakdown, cultural dislocation, racism and discrimination, social disadvantage and poverty (14). Social and emotional wellbeing services are best designed and led by Aboriginal and Torres Strait Islander community controlled organizations and leadership, supported by meaningful partnerships with mainstream organizations across all facets of design and delivery (6). In addition to providing and supporting culturally safe services, youth justice services must support transitional care that facilitates continuity of care from detention to community life (7, 23).

### 2.1. Cultural governance

The IMHIP – youth project includes several levels of oversight by Aboriginal and Torres Strait Islander researchers, clinicians, and community members. It involves cultural leadership provided by an Aboriginal and Torres Strait Islander Cultural Governance Group that includes Aboriginal and Torres Strait Islander project Chief Investigators and Associate Investigators, as well as stakeholders from child and youth mental health service, Aboriginal and Torres Strait Islander Community Controlled Health Organisations and the wider community. The purpose of the Governance Group is to provide Aboriginal and Torres Strait Islander leadership for the design, implementation, evaluation and interpretation of the model of service and research. The Cultural Governance Group’s role is to provide direction, input and advice on the development of the Indigenous Mental Health Intervention Youth Project (IMHIP-Youth; the project). The Cultural Governance Group has been established to ensure cultural leadership, protocols, perspectives and cultural ways of knowing, being and doing are central in all aspects of the project and service development. Key functions include membership on other working groups, providing guidance, advice and recommendations on the development, delivery and evaluation of the project key deliverables.

Additionally, the project aims to employ Aboriginal and Torres Strait Islander researchers and clinicians and provide comprehensive training and mentorship to individuals who are recruited to the project for a culturally-safe working environment.

**Figure 1:**
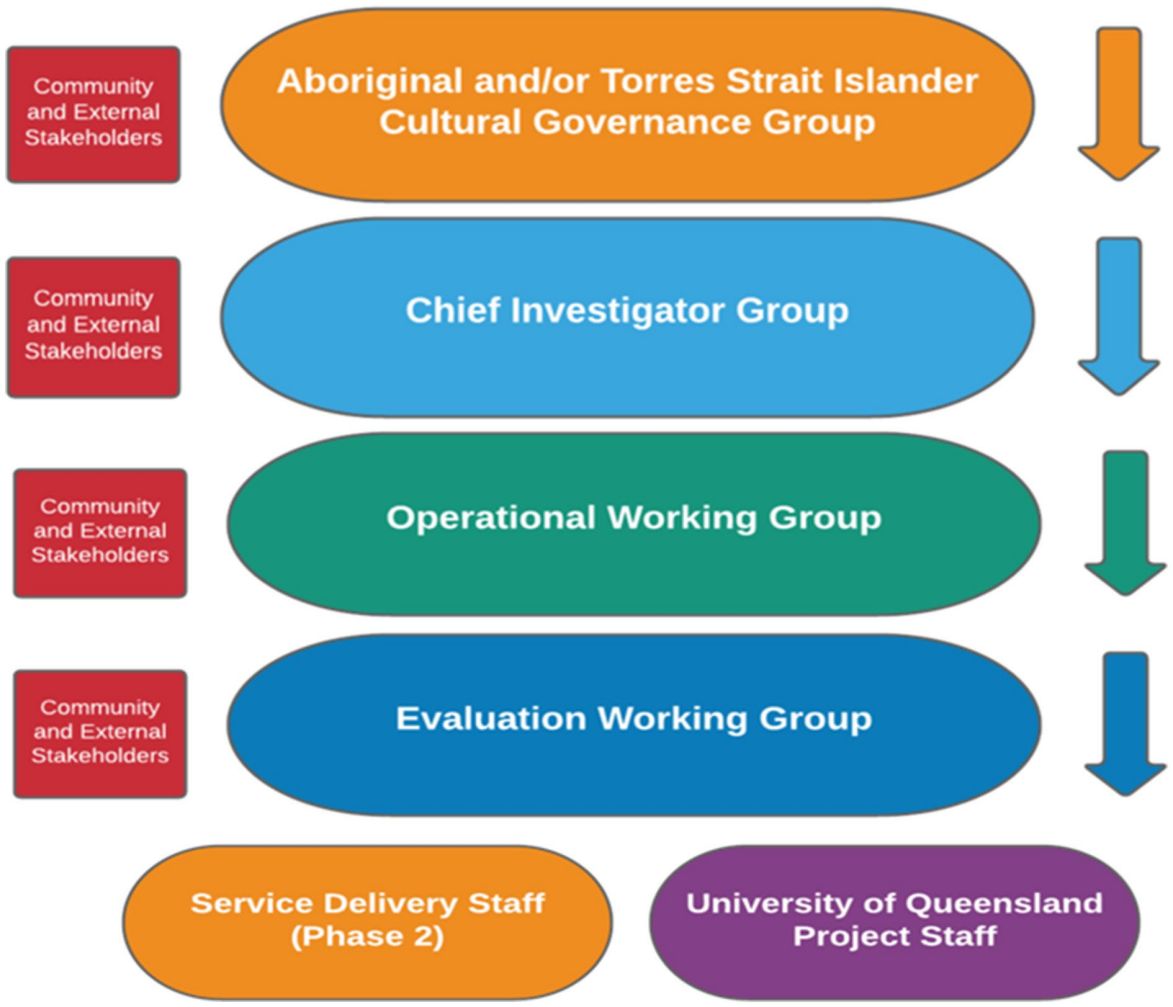
IMHIP-youth project governance structure.

The Cultural Governance structure sits within the broader project structure which includes Chief Investigator Group, Operations Working Group, Evaluation Working Group, project staff and service delivery staff, as shown in Figure 1.

The Governance structure includes:

- The Chief Investigator Group is responsible for the overall delivery of the project, in line with the principles and guidelines of the approved proposal.
- The Operational Working Group provides week to week project guidance by members and is responsible for the day to day management of project activities, including both service and research activities.
- The Evaluation Working Group – guided by the ethics approvals, the Evaluation Working Group is responsible for the intellectual and practical evaluation activities, including data collection, analysis, interpretation, developing and obtaining ethical clearances. This includes collaborating to gather, analyze and report baseline and follow up data to inform service development and understand the reach and effectiveness of the program of work. All project team members have a role in data analysis, interpretation, and reporting.
- Project staff are responsible for delivery of project activities including research, co-design and service development and implementation.

### 2.2. Indigenous mental health intervention program

The IMHIP-Youth model that will be implemented and evaluated builds on an existing social and emotional wellbeing service, the Indigenous Mental Health Intervention Program (IMHIP). This service has been developed by several members of the investigator team and is currently operating in several adult correctional settings in South-East Queensland. IMHIP is Australia’s first Aboriginal and Torres Strait Islander-led social and emotional wellbeing service for Aboriginal and Torres Strait Islander adults in custody. IMHIP has been designed to meet the needs of Aboriginal and Torres Strait Islander adults in custody who experience complex and co-occurring health complications which are intertwined with broader social and emotional wellbeing challenges. IMHIP is committed to Aboriginal and Torres Strait Islander leadership in its development, staffing and model of service, and in ensuring that its evaluation is culturally informed and consistent with wellbeing paradigms.

In 2014, the adult IMHIP was piloted in an adult women’s correctional center (24). In 2017, an expansion of the service was implemented in Queensland’s largest men’s correctional center and into a second women’s correctional center the following year. Involvement in IMHIP is voluntary; The IMHIP applies strengths-based, culturally grounded, trauma-informed approaches and culturally validated assessments and interventions. It aims to provide: early identification; therapeutic interventions for mental health and social and emotional wellbeing needs; and goal setting that informs provision of other interventions in custody and community transitional support for 6 months back to community with an Aboriginal and Torres Strait Islander non-government organization. The IMHIP model also includes a liaison component with adult services including in custody mental health services, corrective services, community forensic mental health services, public and primary mental health services, and other government and non-government support services.

In addition to a Queensland Health in-reach team, the service is complemented by an Aboriginal and Torres Strait Islander non-government organization, whose involvement commences 6 weeks pre-release and 6 months in the community through a service level agreement. The transitional service works collaboratively with the IMHIP in planning transitional needs and supports the handover of care during the last month of custody. The transitional service provides practical support needs (care packages, transport, accommodation, child safety support), ongoing mental health and social and emotional wellbeing support including National Disability Insurance Scheme (NDIS) access where indicted, psycho-education support, cultural links and connections, the provision of accessible services and referrals in multiple geographical locations, and statewide coordination of care, and support and connections for clients returning to communities outside of South East Queensland.

The core elements of the original IMHIP will inform the approach, elements and basis for the IMHIP-Youth model where applicable. Given that the premise of the IMHIP adult was developed from an adult lens through co-design with Aboriginal and Torres Strait Islander adult populations including those with lived experience, families, Elders, communities and government and non-government services that work within the adult populations, elements for applicability for young people will need to be modified through the lens of culturally appropriate childhood stage development, experiences of trauma, length of stay and return to detention rates, and age appropriate transitional service support needs. In particular, the adult IMHIP transitional support component of the program will need to be modified given the age specific needs of the cohort.

#### 2.2.1. Integrated components of IMHIP youth

IMHIP-Youth will be developed through a co-design process, outlined later in this paper. Building on the established IMHIP model, the development of IMHIP-Youth will be staffed by an Aboriginal and Torres Strait Islander workforce and likely comprise the three integrated components of IMHIP:

1. in-reach
2. transitional support
3. collaboration with mental health services in detention.

1. In-reach will be delivered by an Aboriginal and/or Torres Strait Islander workforce and incorporate evidence-based, culturally-validated assessments and e-mental health interventions, including the Stay Strong Plan (25) and an age appropriate version of the Growth and Empowerment Measure (GEM) (26), delivered via Android tablet PCs. The Stay Strong Plan is used in the IMHIP model and is a culturally-adapted brief therapy used among Aboriginal and Torres Strait Islander communities informed by a range of evidence-based therapies including motivational interviewing, problem-solving therapy, positive psychology, solution-focused therapy, and cognitive behavior therapy (25). It is underpinned by an evidence-base that included a literature review, in-depth consultation with Aboriginal and Torres Strait Islander stakeholders, and a randomized controlled trial (25, 27). The Stay Strong App has been evaluated favorably in terms of feasibility, usability, and acceptability in qualitative studies (28, 29). Training will be developed in use of the Stay Strong Plan. IMHIP-Youth will include coordination and provision of culturally safe, trauma-informed care, including identifying and where possible addressing impacts of loss of Aboriginal and Torres Strait Islander cultures, family and community connections.

2. Transitional support will commence before release from detention. This approach both supports the importance of developing relationships but also recognizes that the often brief period in detention offers an opportunity for uptake of services. The transitional service will provide social and emotional wellbeing care, and practical support pre- and post-reintegration to community. This will include liaising with other services to promote cultural connection, as well as assistance regarding accommodation, income/financial issues, employment, education and training, access to culturally safe health and disability services, legal representation, transport, and living skills. IMHIP-Youth will also include a family support service component.

3. Collaboration with mental health services in youth detention. IMHIP-Youth in-reach staff will collaborate with mental health services in youth detention centers to increase the capability for engaging with Aboriginal and Torres Strait Islander young people. The services will establish collaborative care arrangements for clients who require specialist mental health treatment and have unmet social, cultural and emotional wellbeing needs. IMHIP-Youth will facilitate referrals and provide cultural knowledges and recommendations on the young people’s social and emotional wellbeing needs. Collaboration will involve case reviews and sharing information about interventions to ensure services provide complementary and consistent care and support. Workforce development strategies will be included such as cultural safety training, critical self-reflection and development of skills for working with Aboriginal and Torres Strait Islander people including to facilitate access to Aboriginal and Torres Strait Islander Elders and cultural practices.

## 3. Conceptual framework

The methodology for the development of the IMHIP-Youth service (Phase 1) is co-design and the landscape domain of the Ngaa-bi-nya framework. Co-design is a participatory process based on developing strong partnerships between researchers and stakeholders at the outset of a service design initiative. It is underpinned by the principle that those who use, or will have a role in, the service are experts in what they need, and in what works (30). Principles of co-design with Aboriginal and Torres Strait Islander peoples include ensuring that engagement is culturally safe and trauma-informed, and that respect for Aboriginal and Torres Strait Islander research methodologies are embedded from the outset (31). The Ngaa-bi-nya framework is an evaluation framework that aligns with Aboriginal and Torres Strait Islander peoples social and emotional world view and is underpinned by human rights principles and ethical conduct in research with Aboriginal and Torres Strait Islander peoples. The Ngaa-bi-nya framework has four domains which provide prompts for reflection, to stimulate discussion, data collection and analysis (32). Bringing the two approaches (co-design and Ngaa-bi-nya framework) together will enhance the centrality of Aboriginal and Torres Strait Islander peoples voices in the research and service development methodology.

The evaluation will also involve a quantitative component, with baseline data collection will occur in tandem with Phase 1. The evaluation (Phase 2) will further utilize the Ngaa-bi-nya framework for Aboriginal and Torres Strait Islander program evaluation (32).

## 4. Research questions

### 4.1. Phase 1: stakeholder consultation and co-design

In the Ngaa-bi-nya framework, the landscape domain, insofar as it describes important contextual factors relevant to how programs operate, is a domain of information with relevance to both the co-design process and the program evaluation. Consequently, Phase one consultation and co-design will include exploration of the landscape domain.

The landscape domain of the Ngaa-bi-nya framework includes a range of prompts to consider history, whole-of-life, systems and other contextual factors. These are operationalized into Phase one research questions, as follows:

- What is the local history and context of Brisbane Youth Detention Centre (BYDC) and West Moreton Youth Detention Centre (WMYDC)?
- What is the existing services context, within and outside of detention, with respect to which IMHIP-Youth must integrate?
- What is the nature and extent of young people’s participation in current programs and what, if any, barriers exist to engagement?
- What language groups/mobs/regions do Aboriginal and/or Torres Strait Islander young people come from?
- What are Aboriginal and Torres Strait Islander young people’s needs and priorities for a model of service?
- What are the needs and priorities for a model of service, and what will ‘success’ of IMHIP-Youth look like, according to different stakeholders?
- How should this be measured?

### 4.2. Phase 2: IMHIP-Youth evaluation

Phase one will involve quantitative data collection that contributes to a characterization of young people’s need (as identified above), but that will also support the quantitative evaluation of the IMHIP-Youth project, through collecting historical control data. A quasi-randomized controlled trial, with a sequential cohort design (33) was considered the most rigorous quantitative design that was feasible given logistic and ethical considerations of working with a group of young people who experience detention. The research question relevant to the quantitative evaluation of this project fits into the learnings domain of the Ngaa-bi-nya framework:

- Does IMHIP-Youth (i) improve social and emotional wellbeing, (ii) enhance educational and vocational outcomes, and (iii) reduce criminal justice system contact, and reincarceration, of Aboriginal and Torres Strait Islander young people (10–17 years old) who experience detention, in comparison to standard care?

## 5. Study setting

Approximately three-quarters of Queensland’s population lies in its south-east corner, where the detention centers through which this study is being conducted are located. The Brisbane Youth Detention Centre (BYDC) and West Moreton Youth Detention Centre (WMYDC) are located in Wacol, Brisbane, Queensland, Australia. The Yuggera, Jagera and Ugarapul peoples are the Traditional Custodians of the area known as Wacol for over 60,000 years. Wacol is approximately 18 km south west from the Brisbane City (Queensland’s capital city, and most populace city), bounded by the Brisbane River and Wolston Creek. Traditionally the Brisbane River holds significant importance for the Traditional Custodians as a source of native food, animals and environment, hunting grounds and gatherings and ceremonies.

Both centers are established under the *Youth Justice Act 1992* with operational and management by the Queensland Government Department of Children, Youth Justice and Multicultural Affairs. The centers accommodate both females and males aged 10–17 who have been remanded or sentenced to detention for a criminal offence. BYDC has a 162 bed capacity, with WMYDC having a 32 bed capacity with a combination of 194 beds within the two sites at any given time. Young people placed in BYDC may be from a wide geographical location including Western and Southern Queensland communities to the Queensland boarder including boarder towns and communities with Queensland and other States and Territories and north to Central and Western Central Queensland communities.

BYDC and WMYDC provide young people with a routine day with activities ranging from education, training, developmental, recreational and cultural programs. In addition, young people have facilitated visits from families and care-givers of young people, legal support and advocacy, and community members and Elders.

## 6. Methods

This project has received ethical clearances and research approvals from the Children’s Health Queensland Human Research Ethics Committee (HREC/20/QCHQ/70612), the University of Queensland (2021/HE001359), and Department of Youth Justice and Multicultural Affairs.

### 6.1. Stakeholder consultation and co-design

Phase one will involve stakeholder consultation that will contribute to the co-design of an IMHIP-Youth model. Structured consultations with stakeholders will seek to answer several of the Phase one research questions, pertaining to the landscape domain.

Consultation and co-design with community is a commitment to establishing and using shared language, openness of information and addressing power imbalances with all experts throughout the process. The opportunity is co-design through multiple perspectives with differing views acknowledged and accepted with all working in partnership to develop, prioritize, implement and evaluate outcomes. Those involved throughout all stages are acknowledged and provided with opportunities to opt in and out of the process. The project team’s role is to listen, assess and integrate the information shared in a transparent way. In this way, consultation within a co-design framework differs from earlier models of community consultation in that it will go beyond simply obtaining information to analyze and inform decision making, within pre-specified parameters, to instead ensure that concerns and aspirations are consistently understood and considered. Co-design will be a meaningful and authentic process, that shares power and builds partnerships to co-create health services.

Co-design and consultation will be undertaken with relevant stakeholders, including:

- Aboriginal and Torres Strait Islander young people who have experienced detention
- Aboriginal and Torres Strait Islander Elders
- Families and carers of Aboriginal and/or Torres Strait Islander young people who experience detention
- Relevant Government Departments
- IMHIP staff
- Non-Government Organization providers who work with justice involved young people
- Staff who deliver health services within youth detention centers and once in community.

### 6.2. Co-design with adult stakeholders (adults, 18 years and over)

Focus groups, meetings, panel discussions, and one-on-one consultations will occur with Elders and other stakeholders, in order to understand elements of the *Landscape domain* through understanding needs, mapping historical and contemporary contextual factors, mapping the current services context, and envisioning what success would look like. This will ensure that a comprehensive understanding of stakeholder perspectives is gathered. It is envisaged that 6–12 individuals will participate per stakeholder group to gather diverse views. A multi-stakeholder workshop will also be held to summarize findings of the consultation and develop consensus around the model of service to be implemented and finalize details of its evaluation. This is a critical step to informing service design. Specific stakeholder views (i.e., excerpts of narratives or transcribed audio) will not be published as a research output.

All stakeholders who are engaged will be supplied with information sheets describing the nature of the study and their participation or directed to the project website, www.IMHIP-Youth.org, which will be regularly updated and provides a comprehensive repository of information on the project. Verbal consent to participate will be sought, but participants will not be asked for signed written consent forms, due to the fact that the primary purpose of this engagement is consultation and data gathering with respect to services and systems but is not to collect the opinions or attitudes of individuals themselves for the purposes of research on individuals. Collecting written consent forms would be out of keeping with norms of co-design and consultation processes and could restrict or skew the engagement process and/or flow of ideas.

### 6.3. Engagement and co-design with Aboriginal and Torres Strait Islander young people (<18) who have experience of detention

Processes for recruiting and gathering consent from young Aboriginal and Torres Strait Islander peoples are described below.

#### 6.3.1. Eligibility

Aboriginal and Torres Strait Islander young people will be eligible to participate in Phase one of this study, if they:

- Identify as an Aboriginal and/or Torres Strait Islander person
- Were or are aged 10–17 years old at the time of their offence date for which they entered into detention, during the recruitment period
- Entered one of the two Brisbane youth detention centers during the recruitment period, or left detention in the 12 months prior
- For those in detention only, have been received into detention within the past 7 days OR are already in detention, but not received in the past 7 days, in which case they are eligible to participate in an initial interview, but will not be followed up, and
- Have sufficient understanding, intelligence and maturity to appreciate the nature, consequences and risks of consenting to participate in the study and the alternatives, including the consequences of not consenting to participant.

#### 6.3.2. Consent

Prior to commencing recruitment, all staff from the above-mentioned services and organizations who will be assisting in recruitment will receive a project orientation, through an introductory presentation by project team members. This will include an overview of the aims of the project, discussion of informed consent processes, and training to ensure that appropriate cultural protocols are followed in recruiting and engaging young people and their parents. The engagement with parents and care-givers is an important part of the consent process as consent by young people is complex due to a number of factors including age, developmental stage, capacity, language and comprehension to what they are providing consent for. To safeguard the young person’s and family rights, and to honor co-design, parents and care-givers will be provided with the same level of information and opportunity to ask questions to ensure informed consent is given throughout the process. Where possible, individuals involved in recruitment of participants will be Aboriginal and/or Torres Strait Islander people.

When determining a young person’s capacity to provide free and informed consent to participate, researchers will be guided by Queensland Health Guidelines on the assessment of a child’s capacity to provide consent, which include consideration of the following:

- the age, attitude and maturity of the child or young person, including their physical and emotional development
- the child or young person’s level of intelligence and education
- the child or young person’s social circumstances and social history
- the nature of any condition the child or young person may be diagnosed with
- a young person aged between 16 and 18 is most likely able to consent
- a young person aged between 14 and 16 is reasonably likely to be able to consent
- a child under the age of 14 may not have the capacity to consent

All eligible participants will be approached during the period of recruitment for Phase one (planned for 8 months, beginning in July 2021), or until 110 individuals are consented. Participants and their parents or guardians will be asked to provide consent for:

- primary qualitative and quantitative data collection (described in data collection section, below)
- consent to be contacted by researchers in the future to undertake further data collection relating to the project’s evaluation, and
- consent for the operating department of the youth detention centers to provide identifying details, including aliases, to an independent government statistical organization to undertake data linkage, that will occur during Phase 2 of the research. Data linkage will be to health, education custodial datasets.

#### 6.3.3. Recruitment

For Phase one, Aboriginal and Torres Strait Islander young people who have experienced detention will be recruited through:

1. Forensic Child and Youth Mental Health Service in detention and community
2. Brisbane Youth Detention Centre or West Moreton Youth Detention Centre
3. Participating community-controlled organizations

##### 6.3.3.1. Recruitment via forensic CYMHS

**Figure 2:**
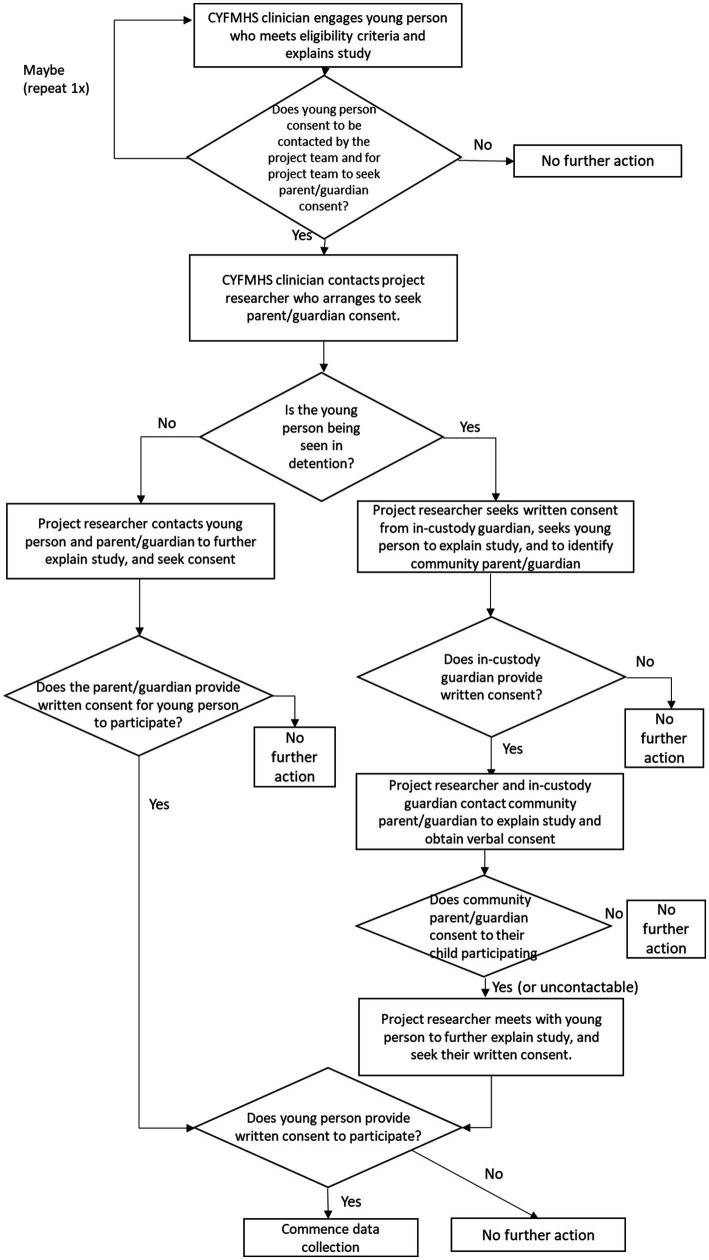
Recruitment and consent process via Forensic CYMHS.

The recruitment and consent process via the existing Forensic Child and Youth Mental Health Services is given in Figure 2. Individuals meeting study eligibility criteria will be engaged by clinicians who have received orientation and training, to explain the project to the prospective participant in written and spoken form. The young person will asked if they would be interested in being contacted by a researcher to obtain consent from them and their guardians to participate. The young person will be informed that they can withdraw this consent at any time.

If an individual gives consent for a project researcher to seek parent or guardian consent, Forensic CYMHS will contact the project researcher to provide contact details for the young person’s parent or guardian. If the participant is in custody, the in-custody guardian will be contacted to advise them of the participant’s interest and to plan to meet with both parties to explain the study. When the young person and in-custody guardian have consented, researchers and the in-custody guardian will contact the relevant community parent/guardian, to obtain verbal consent for participation. If the community parent/guardian does not consent, then the interview will not proceed. If the community parent/guardian cannot be readily contacted (i.e., two unsuccessful attempts are made over a 24 h period), the interview will proceed on the basis of the consents obtained. The researcher will contact the community parent or guardian to obtain written consent if the young person is not in custody at the time of approach.

##### 6.3.3.2. Recruitment via youth justice pathways, within Brisbane Youth Detention Centre and West Moreton Detention Centre

**Figure 3:**
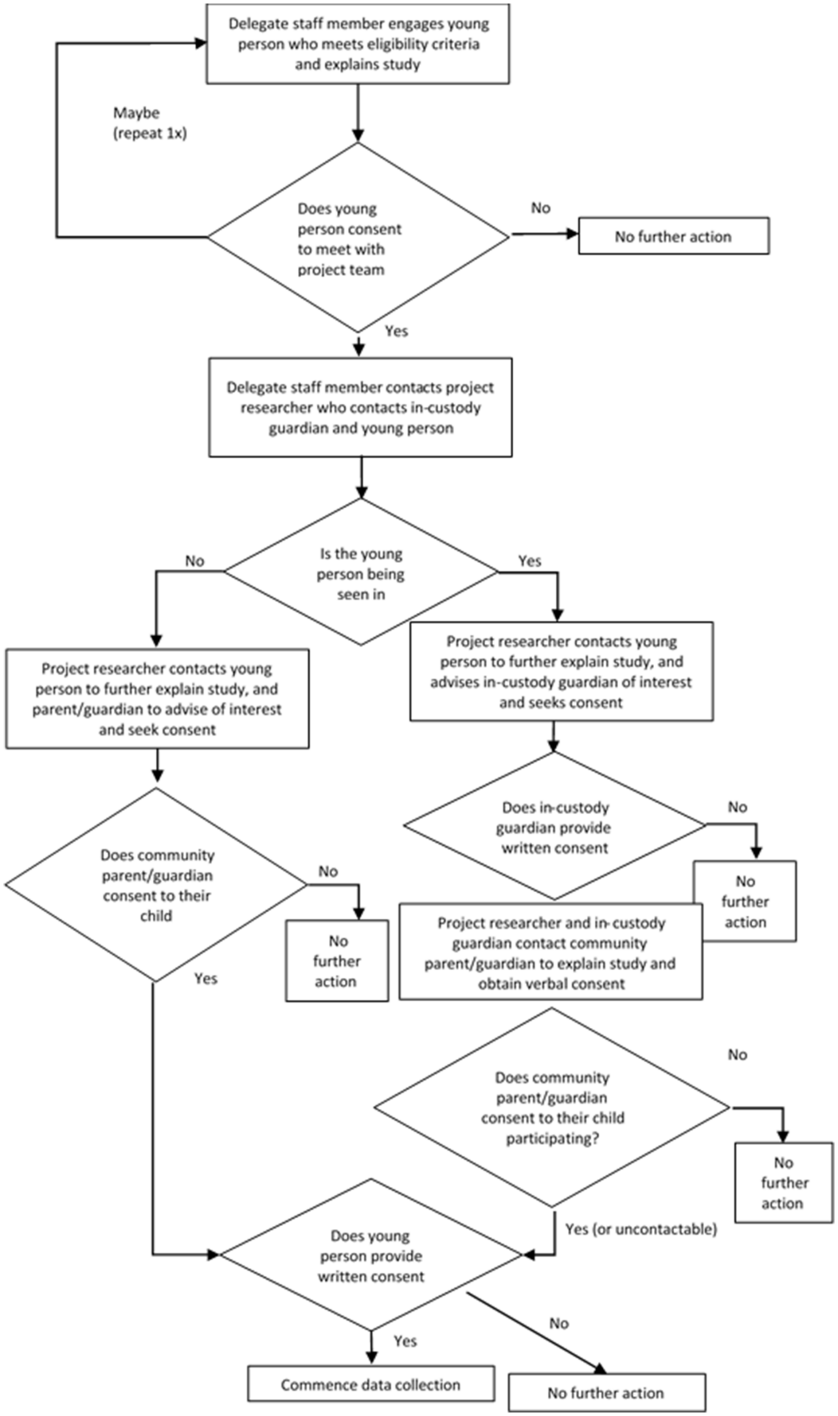
Recruitment via Brisbane Youth Detention Centre and West Moreton Detention Centre pathways.

The recruitment and consent processes within the detention centers will follow a similar process to recruitment via Forensic CYMHS. This recruitment process is provided in Figure 3. Individuals meeting study eligibility criteria will be engaged by a delegated staff member working in the centers who has received orientation and training, to explain the project to the participant in written and spoken form. The young person will asked if they would be interested in being contacted by a researcher to obtain consent from them and their guardians to participate. The young person will be informed that they can withdraw this consent at any time.

If an individual gives consent to be contacted and for a researcher to seek guardian consent, the delegate will contact the project researcher to provide contact details for the young person’s guardian. If the participant is in custody, the in-custody guardian will be contacted to advise them of the participant’s interest. When the young person and in-custody guardian have consented, researchers and the in-custody guardian will contact the relevant community parent/guardian, to obtain verbal consent for participation. If the community parent/guardian does not consent, the interview will not proceed. If the community parent/guardian cannot be readily contacted (i.e., two attempts are made over a 24 h period), the interview can proceed. The researcher will contact the community parent or guardian to obtain written consent if the young person is not in custody at the time of approach.

##### 6.3.3.3. Recruitment via Aboriginal and Torres Strait Islander community controlled organizations

**Figure 4:**
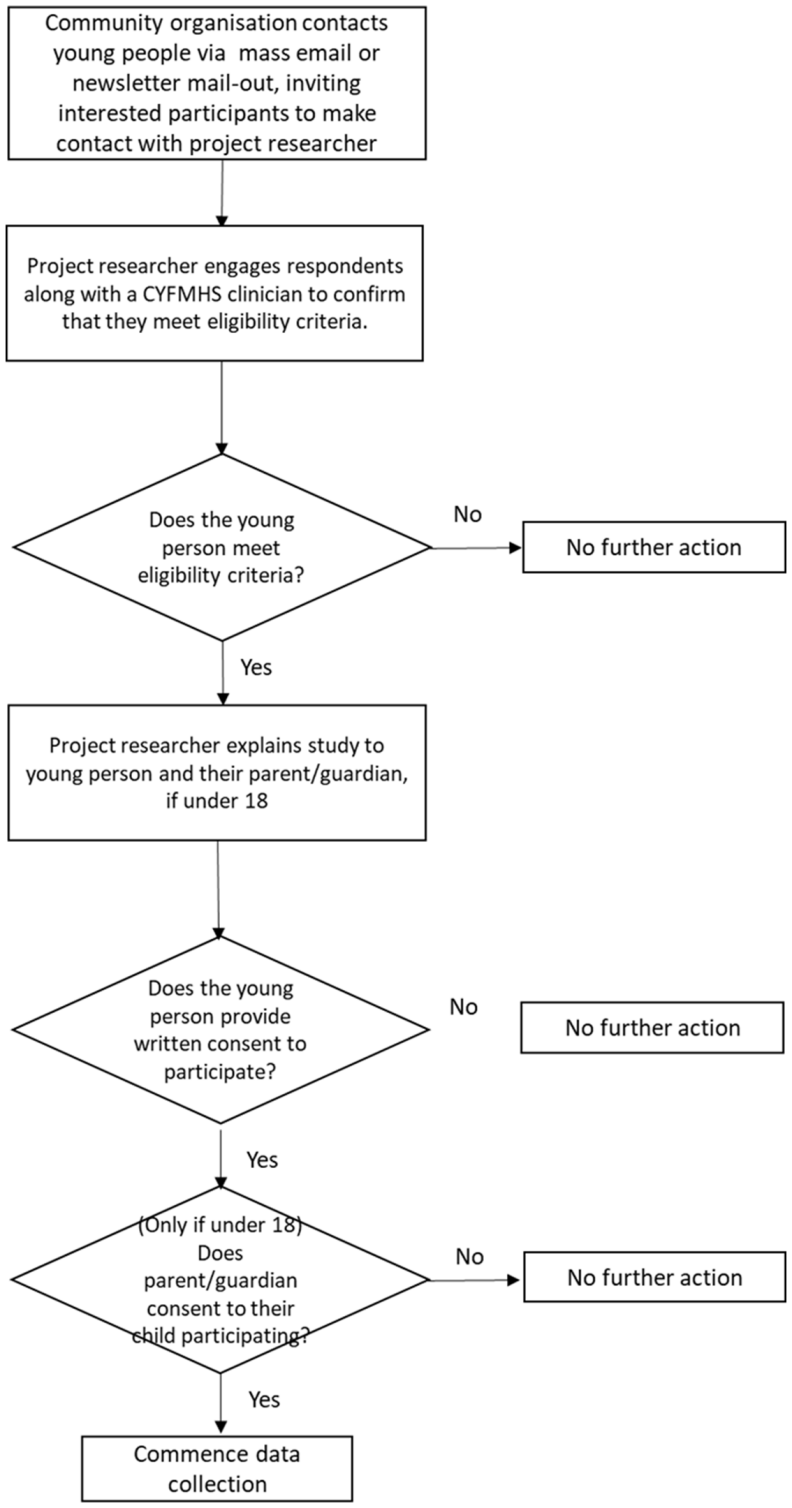
Recruitment and consent via community controlled organizations.

In addition to recruitment within detention settings, some recruitment will occur among individuals who are in the community (Figure 4). This will be critical to informing the design of the transitional service, by engaging and hearing the perspectives of individuals who have left detention and are in the community. Recruitment via Aboriginal and Torres Strait Islander Community Controlled Organizations will occur via online channels (e.g., mass emails or newsletters). Project information, an invitation to participate in the research, information regarding the nature of the project and participation information will be provided at this time.

Individuals who contact the project team via this channel will be followed up as soon as is practicable by a member of the research team. The researcher will explain the study to the young person and their parent or guardian, and seek their consent. The process of seeking consent could occur simultaneously, if parents and children agree and are available at the same time; otherwise it will occur sequentially. A CYFMHS clinician will join the project team member for the purposes of assessing that the prospective participant has capacity to consent. However, it should be noted that we anticipate that many individuals recruited via this pathway will be 18 or 19 years of age at the time of interview, and therefore parent or guardian consent will not be required.

#### 6.3.4. Request for additional de-identified linked data for young people who experience detention, where a waiver of consent will be sought

The unforeseen events of the COVID-19 pandemic have had several impacts on young people in detention. It is unknown what impacts these will have on trends in social and emotional wellbeing, or educational, vocational or criminal justice outcomes. In order to improve the evaluation in the context of unknown COVID-19 related variation, we are requesting linked data for two historical cohorts, based on entrants into youth detention for two 8 months periods in the 2 years prior to the recruitment phase of the project. It is not feasible to contact or obtain consent from these individuals, and therefore a waiver of consent is requested for these cohorts.

### 6.4. Data collection

#### 6.4.1. Aboriginal and Torres Strait Islander young people with experience of detention

The target sample size for Phase one recruitment is 110 individuals, comprising 80 individuals who are received into detention within the last 7 days, during the recruitment period, and 30 individuals who are in the community, but had an experience of youth detention in the 12 months prior or who are in detention, but have been in detention for more than 7 days during the period of data collection. Individuals will be recruited over an approximate 8-month period commencing in July, 2021.

All young people who agree to participate in Phase one of this research will be invited to participate in an initial interview. The 80 individuals who are recruited within 7 days of detention will also be invited to participate in a series of follow-up interviews that will provide baseline data for quantitative comparison. Planned follow up interviews will take place at 30 days following the initial interview, 6 months following the initial interview and 12 months following the interview. Interview schedules are described below.

#### 6.4.2. Initial interview

Interviews will take place at a time that does not conflict with structured programs that the young person should be engaging with in detention. The initial interview will be a one-on-one interview with an experienced Aboriginal and/or Torres Strait Islander interviewer, trained additionally for IMHIP-Youth. Interviews are a supportive respectful way to hear young people’s perspectives. They have also been chosen as a data collection method due to identified limitations to open disclosure among young people in detention in group-based settings as well as because of the detail required about topics in relation to which data are gathered. Culturally-safe, age-relevant questions, including life journey drawing and scenarios, will be used to engage with young people within the context of, what Bessarab and Ng’andu (34) have described as, “research topic yarning.” Yarning describes an Aboriginal form of communication and conversation for building respectful relationships within a culturally safe space. The ‘yarn’ forms the basis for relational interactions, knowledge and information sharing, giving or finding for a collective and collaborative understanding. Importantly, yarning refers to the establishment of a relationship with participants prior to gathering stories. Interviews will seek to understand young people’s needs and priorities in the context of their narratives of past, present, and future (34).

Interview will take approximately 45 min, to ensure that young people do not experience undue fatigue. Young people will be able to cease the interview at any time and for any reason, and they will have the option to recommence and complete the interview over two sessions, if they wish. They are also able to withdraw consent to participate at any time, by notifying the researcher they are engaging with or contacting a project team member (contact details will be provided on information sheets).

##### 6.4.2.1. Measures and interview guide

The interview will begin with the Growth and Empowerment Measure (GEM) (26). The GEM was developed by and validated for Aboriginal and Torres Strait Islander Australian adults, however, it has been successfully used among young people. The GEM is a 26-item self-report measure using culturally-relevant scenarios and language to understand social and emotional wellbeing, connections with others and empowerment. The GEM has been modified for this study, modifications include: simplification of language, and modification of questions to reflect the age of participants (e.g., reference to having children will be removed from response formats, and references to employment and training will be reworded to incorporate participate in schooling). The GEM-Youth, will be piloted and validated at the beginning of the project. The GEM-Youth instrument (as well as the K-10) will be completed on paper or tablet, with the trained interviewer actively participating in the young person’s completion of these instruments, to ensure that questions are understandable and understood by participants, including those who may have literacy issues, and that answers given are an accurate reflection of the young person’s views or attitudes.

After the GEM the interviewer will administer the Kessler-10. K-10 is a widely used measure of 30-day psychological distress, with well-established validity and reliability. The K-10 has been culturally validated with Aboriginal and Torres Strait Islander people.

Additionally, identifying data and socio-demographic data will be recorded on:

- Name, including any aliases
- Date of Birth
- Current Address
- Child Protection Status (No Child Protection Order, Historical CPO, Intervention with Parental Agreement, Temporary Custodial Child Protection Order, Long Term Guardianship Child Protection Order)
- Sex/Gender (Male, Female, Other)

The interview will end with supportive reflection on the young people’s story through a narrative interview that will explore topics such as:

- What led them to being in detention – the factors, not crime
- What their experience of detention is (or was) like, what programs they participate(d) in, what they are hoping to achieve
- How they feel about their health and wellbeing, including social and emotional wellbeing, and any mental health concerns
- What they hope to do when they leave detention (or what they have done since leaving detention), what concerns they might have for their future, and longer-term aspirations they may have including for education and vocation.
- What they would like from a service that was designed to help improve their social and emotional wellbeing, and what success would look like to them.

Six alcohol and drug questions will also be asked, as follows:

- Do you drink alcohol? (Yes/No)
  - If Yes
    ■ are you worried or concerned about your drinking? (Yes/No)
    ■ have your family or friends said that they are worried or concerned about your drinking (Yes/No)
- Do you use drugs? (Yes/No)
  - If Yes
    ■ Are you worried or concerns about your drug use? (Yes/No)
    ■ Have your family or friends said that they are worried or concerned about your drug use? (Yes/No)

#### 6.4.3. Interviews at 30 days, 6 and 12 months following reception into detention

Young people (*N* = 80, target) who are recruited in detention will be asked if they consent to be followed up at 30 days following the interview, and again at 6 months and 12 months following the interview.

The interview that takes place at 30 days, 6 months and 12 months will be a brief ‘check-in’, principally for the purposes of gathering GEM and K-10 measures, as well as alcohol and drug questions.

#### 6.4.4. Data linkage

At the initial interview, consent will be sought from participants and their guardians to undertake data linkage to a range of records, for the purposes of understanding the profile and needs of participants, and assessing outcomes. Data sources to be linked are:

- Health service usage data
- Ambulance service usage data
- Government department holding data related to young people who have experienced detention

### 6.5. Analysis

#### 6.5.1. Quantitative analysis

Quantitative data collected from Phase one will be analyzed using descriptive statistics to provide a profile of the cohort. Inferential statistics (ANOVA, Chi-squared tests, linear regression) will be used to compare sociodemographic factors and measures of mental health, and social and emotional wellbeing, and educational and criminal justice outcomes, across the three recruitment channels. Associations between sociodemographic factors and mental health and wellbeing will also be explored. Latent class analysis and/or group-based trajectory modeling will be used to analyze the trajectory of outcomes over time and to explore whether there are meaningful subgroups within the population.

GEM-Youth and K-10, along with alcohol and drug questions, collected from young people in detention (i.e., recruitment via child and youth mental health service and the Aboriginal and Torres Strait Islander unit), as well as socio-demographic data, will be used as comparison with the intervention cohort in Phase 2. Identifying data (Name, Date of Birth, Address) will be used for the purposes of data linkage.

The GEM-Youth will be piloted during the first five interviews of this study. Any issues identified with language, understanding or logistics of this tool will be discussed among investigators Williams, Penny, Meurk and Wittenhagen, and a decision will be made as to whether modifications to the tool are required. Following completion of initial interviews, the GEM-Youth will be validated, against the K-10. The methodology will follow the approach taken by Haswell et al. (26), in their Psychometric validation of the GEM. The psychometric properties and internal structure of the GEM (Adult) was examined against the K6 and K6 + 2, utilizing Pearson’s correlations, Cronbach’s alpha, and Principal Components Analysis.

#### 6.5.2. Qualitative analysis

Interviews will be audio-recorded and stored in a re-identifiable form and be partially transcribed by project team members. Drawings produced during the initial interview will be copied with permission of the young person and the original returned if they wish.

Qualitative data will be analyzed using an inductive thematic approach, structured within the framework of the *Ngaa-Bi-Nya* landscape prompts (35). Analysis will include ongoing consultation with the Cultural Governance Group throughout, to ensure that culturally specific meanings and nuances are not missed and that findings are presented in a culturally safe way. Data presented in publications will be assigned a pseudonym, with no identifying details accompanying excerpts or artworks that may appear in publication.

## 7. Remuneration

Participants will be remunerated for their time with a $20 voucher (e.g., Rebel Sport, Kmart, Cotton On or City Beach) (initial, 30 days, and 6 months), except for the last payment (12 months), where they will receive $30. Participants who complete data collection at one time point over two sessions, will receive their payment on completion of all data collection activities for that time point. In line with Aboriginal and Torres Strait Islander ways of doing, remuneration processes align with the principle of reciprocity when sharing personal stories. Given the relatively low risks of participation in this study, and processes we have in place for managing wellbeing risks, we believe that remuneration is both ethically and culturally important (36).

Participants will also be offered the opportunity of submitting something to appear on the project website^1^ e.g., a story, artwork, recipe or joke. These contributions will be anonymous and an opportunity, only if the young person is interested.

### 7.1. Other stakeholders (≥18 years)

As above, individual level data will not be collected from other stakeholders in Phase one for the purposes of research, and these participants will not be remunerated. However, where possible reciprocity will be shown through provision of opportunities for training or refreshments being provided at events. If members of the community are asked to facilitate any engagement activities (e.g., as a Chair), they will be remunerated for their time in line with relevant university policies.

## 8. Publication and dissemination

Findings from Phase one will be used to inform the development of a provisional model of service for a social and emotional wellbeing intervention that will be implemented in Phase two. Additionally, quantitative findings will be published in reports, academic articles and conference presentations. Findings from interviews undertaken with young people will be produced into a booklet, comprising narratives and artwork, that can be gifted back to project participants and distributed among other project stakeholders as well as be used in publications.

## 9. Cultural and ethical considerations

### 9.1. Cultural integrity

To ensure cultural integrity of the project, process and outcomes, Aboriginal and Torres Strait Islander ways of being, knowing and doing have been embedded into all aspects of the research processing including the co-design. Application of Aboriginal and Torres Strait Islander principles and practices govern processes including integrity, inclusivity, respect, reciprocity, participative, outcomes focused and promotes self-determination to challenge system barriers including systemic racism. Cultural integrity includes holistic considerations of the individual young person, family and community, and kinship structures, as well as protocols for service design.

As identified earlier, the governance structure and roles of the Aboriginal and Torres Strait Islander Cultural Governance Group works with and provides input into all project activities with the other project groups [Chief Investigator Group (CIG), Evaluation Working Group (EWG), Operations Working Group (OWG)], broadly, and specifically with respect to the values of spirit and integrity, cultural continuity, equity, reciprocity, respect, and responsibility.

A key role is to ensure that the work reflects and acknowledges the broader policy and research context regarding development of mental health and social and emotional wellbeing services including, but not limited to:

- The Cultural Respect Framework for Aboriginal and Torres Strait Islander Health (37)
- A National Strategic Framework for Aboriginal and Torres Strait Islander Peoples’ Mental Health and Social and Emotional Wellbeing (6)
- Values and Ethics: Guidelines for Ethical Conduct in Aboriginal and Torres Strait Islander Health Research (36)

This includes monitoring and providing feedback to the CIG, OWG and EWG on these matters, specifically to monitor and identify cultural risks and measures including development of data sovereignty to ensure culturally safe data collection, analysis and presentation. The data is considered through a cultural community lens to ensure that all data is not mis-interpreted or mis-represented.

### 9.2. Risks

#### 9.2.1. Risks of disempowerment or discomfort

Research in correctional settings can be an empowering experience for participants in spite of limitations because of the location, context and perception of research being invasive. The data collection strategy for this project has been developed in consultation with Aboriginal and Torres Strait Islander investigators and stakeholders, to ensure that it is trauma-informed and empowering. Use of the GEM will ensure that the interview follows a strengths-based approach. A supportive interview context within which to manage risks will be actively constructed for IMHIP-Youth, managed in a variety of ways including:

- Ensuring the research is explained and recruitment is undertaken by an Aboriginal or Torres Strait Islander staff member or staff member experienced in engaging well with young people,
- Provision of training to all staff who will support recruitment, including training in study design and purpose, cultural protocols for engagement and obtaining consent,
- Inclusion of parents or community guardians in consenting process, including in-custody, and
- Provision of age and culturally appropriate written project materials, as well as explanation of the study

#### 9.2.2. Consent risks

Several mechanisms are in place to ensure that young people have capacity to provide informed consent and that their parents and guardians, both in-custody and community, are engaged and have the opportunity to provide their consent to the young person’s participation. These measures are as follows:

- Eligibility to participate based on capacity to consent will be assessed by child and youth mental health service clinician or a youth detention Aboriginal and/Torres Strait Islander staff member, to ensure that the young person has sufficient understanding, intelligence and maturity to appreciate the nature, consequences and risks of consenting to participate in the study and the alternatives, including the consequences of not consenting to participate.
- Young people will be provided with both written project documents, that are culturally and age appropriate, as well as having the project and the nature of their participation explained to them verbally.
- Parents and/or guardians will be engaged in the consenting process, irrespective of whether the young person is being engaged in-custody or community.

#### 9.2.3. Risks due to sensitive questions

Young people who experience detention are more likely than young people in the community to have experienced traumatic life experiences that may be recounted in the process of a narrative interview. Consequently, there is a risk that engagement could cause distress to participants. To manage any risk of harm, interviewers will undergo thorough training in cultural protocols and interviewing techniques as well as trauma informed care. Interviewers will also be provided supervision by a senior clinician. Done well, participating in a narrative interview with a trained interviewer who engages in deep-listening, may be therapeutic and in this project, the interview will follow the completion of the GEM, a trauma-informed strengths based tool. A senior child and youth forensic mental health psychiatrist will be on-call for the duration of the project, to provide advice where a researcher or clinician raises concerns about acute mental health deterioration or a participant’s safety, and to facilitate an appropriate care response. Any emergent distress or concerning statements (e.g., disclosures of suicidality) will be taken seriously, and the researcher will contact relevant Youth Justice staff and the senior child and youth mental health psychiatrist, or his appointed delegate, to seek advice, undertake safety planning, and ensure that appropriate follow-up is provided. If such an event occurs, a review will take place through the Operational Working Group, to identify any issues or learnings relating to the conduct of this research.

#### 9.2.4. COVID-19 risks

The COVID-19 pandemic is having unforeseen and unforeseeable impacts on daily activities, globally. It is likely that COVID-19 will impact upon this project, in terms of its impacts on social and emotional wellbeing, delays due to periodic restrictions on face-to-face meetings, potential impacts on comparator data collection, and rapid shifts in the services landscape. Impacts are already being addressed through the addition of retrospective time points, to assist in disentangling impacts of COVID versus the proposed intervention on social and emotional wellbeing. If required, the project team will substitute the planned face-to-face stakeholder workshop with an online forum, and if/when face-to-face recruitment is not possible, the project team will undertake video-link engagement participants.

#### 9.2.5. Staff risks

Some research staff will be involved in face-to-face data collection that they may find distressing. Some staff will be required to travel offsite for data collection. Any distress will be managed through debriefs with supervisors, with services readily available through The University of Queensland, if required. Risks related to offsite work will be minimized by ensuring that staff do not travel alone. Offsite data collection will be arranged in suitable locations that ensure researcher safety (e.g., rooms in local health services).

## Author contributions

PD, CM, MeW, MS, LW, and SK contributed to the development of the methodology. PD, CM, and MaW contributed to the original drafting of the manuscript. EH provided oversight leadership and responsibility for the protocol development. CM was responsible for the management and coordination of the protocol development. PD, CM, MeW, MaW, MS, SH, SS, JS, SK, and EH were responsible for the acquisition of the financial support for the project leading to this publication. IMHIP Youth Cultural Governance Group provided cultural oversight, knowledge and input into the protocol development. All authors reviewed and approved the intellectual content of the manuscript and contributed to conceptualization of the manuscript.

## Footnotes

^1^ www.IMHIP-Youth.org
